# Meta-Transcriptomic Analysis Reveals Novel RNA Viruses in *Hippocampus erectus*

**DOI:** 10.3390/v15030772

**Published:** 2023-03-17

**Authors:** Fan Zhang, Zhihao Ren, Xiaomeng Guo, Yiting Wang, Fanzeng Meng, Weifeng Shi, Xinping Wang, Xuan Dong

**Affiliations:** 1 College of Marine Science and Biological Engineering, Qingdao University of Science and Technology, Qingdao 266042, China; 2 Yellow Sea Fisheries Research Institute, Chinese Academy of Fishery Sciences, Laboratory for Marine Fisheries Science and Food Production Processes, Pilot National Laboratory for Marine Science and Technology (Qingdao), Key Laboratory of Maricultural Organism Disease Control, Ministry of Agriculture and Rural Affairs, Qingdao Key Laboratory of Mariculture Epidemiology and Biosecurity, Qingdao 266071, China; 3 Key Laboratory of Animal Epidemiology and Zoonosis, Ministry of Agriculture, College of Veterinary Medicine, China Agricultural University, Beijing 100193, China; 4 College of Fisheries and Life Science, Shanghai Ocean University, Shanghai 201306, China; 5 Shanghai Institute of Virology, Shanghai Jiao Tong University School of Medicine, Shanghai 200025, China

**Keywords:** virome, seahorse, *Hippocampus erectus*, transcriptomic, RNA virus

## Abstract

Lined seahorse, *Hippocampus erectus*, is an important aquatic animal due to its medicinal and ornamental purposes. However, our understanding of the viral spectrum in *H. erectus* is still limited. Here, we studied the viruses in *H. erectus* using meta-transcriptomic sequencing. A total of 213,770,166 reads were generated and assembled de novo into 539 virus-associated contigs. Three novel RNA viruses from the *Astroviridae*, *Paramyxoviridae*, and *Picornaviridae* families were finally identified. In addition, we identified a strain of nervous necrosis virus from *H. erectus*. In particular, the unhealthy group showed a higher viral diversity and abundance than the normal group. These results revealed the diversity and cross-species transmission of viruses in *H. erectus* and highlighted the threat of viral infections to *H. erectus*.

## 1. Introduction

With the extensive deployment of high-throughput sequencing (HTS), our understanding of the virosphere in a lot of species has dramatically expanded [1,2]. These also include many aquatic animals, such as fish and crustaceans [3,4,5].

Seahorses are important marine economic animals due to their medicinal and ornamental purposes [6]. Seahorses have a very specific morphology compared to typical fish species, such as a horse-like head, a tubular mouth without teeth, a male brood pouch, and the absence of a caudal and ventral fin [7]. Seahorses are distributed worldwide, including both tropical and temperate regions [8]. The lined seahorse, *Hippocampus erectus*, belongs to the family *Syngnathidae*, genus *Hippocampus*. The name—lined seahorse—was derived from the fine white lines covering the head and body, and it is native to marine waters reaching from Nova Scotia, Canada in the north to Venezuela in the south [9]. Unfortunately, the population size of wild *Hippocampus erectus* has declined dramatically in recent years due to overfishing, and it has been listed on the IUCN Red List of Threatened Species [10]. Because lined seahorses are more suitable for captive breeding than other traditional seahorses [11], the breeding population has grown rapidly in China in recent years [12]. However, one of the major threats to seahorse cultivation is epidemic disease, resulting in severe economic losses.

In this study, we characterized the viral spectrum in three unhealthy and three normal *H. erectus* using transcriptomic sequencing. The newly identified viruses belonged to the *Astroviridae*, *Paramyxoviridae,* and *Picornaviridae* families, which expanded the viral diversity of *H. erectus* and broadened the host range of nervous necrosis virus (NNV).

## 2. Materials and Methods

### 2.1. Sample Information

In this study, two batches (*n* = 6: 3 unhealthy and 3 normal) of lined seahorses were collected from Shandong Province, China, in July and November 2019, respectively (Table 1). The body surface of the unhealthy seahorse darkened and died within 7 to 15 days after the onset of symptoms, with a mortality rate of 100%.

### 2.2. RNA Extraction and Sequencing

RNA extraction, library construction, and transcriptome sequencing were performed as previously described [13]. The total RNA used for transcriptome sequencing was extracted from six (three diseased and three normal) samples with TRIzol reagent (Invitrogen, Carlsbad, CA, USA). A total of 50 mg of the tissue was homogenized by adding 1 mL of TRIzol reagent, according to the manufacturer’s instructions, followed by RNA purification using chloroform and isopropanol. Each sample was sequenced separately using pooled tissues in order to better discriminate the differences in the viral spectrum between samples (Table S1). The RNA used for Sanger sequencing was extracted from 20 mg of liver tissue from the sample FRRL190067412.

### 2.3. Virus Discovery and Confirmation

Adaptor trimming and quality control of raw data was performed using the Fastp program (version 0.21.0) [14]. Clean reads were assembled de novo using Trinity (version 2.5.1) [15]. All the assembled contigs were compared with the non-redundant protein database and the reference virus database using BLASTx, with an *E*-value threshold set at 1 × 10^−5^. All potential viral contigs were identified, and reads were then mapped onto them using Bowtie 2 (version 2.4.1) [16]. Finally, consensus sequences were obtained by continuously merging short contigs to form longer viral contigs using Geneious [17].

### 2.4. RT-PCR

Since the genome structure of Hippocampus erectus astro-like virus 1 (abbreviated as HeAstV1) is distinct from other members of the family *Astroviridae*, RT-PCR of the original sample was performed using the PrimeScript^TM^ One Step RT-PCR Kit Ver.2 (TaKaRa, Beijing, China), with primers designed based on the obtained consensus sequence to further confirm the viral genome (Table S2). RT-PCR was initiated at 50 °C for 30 min and 94 °C for 2 min, followed by 30 cycles of 94 °C for 30 s, 60 °C for 30 s, and 72 °C for 1 min. If the concentration of the RT-PCR product of the first round was low, nested PCR was performed using Premix Taq^TM^ (TaKaRa, Beijing, China). The reaction conditions for nested PCR were 30 cycles of 98 °C for 10 s, 60 °C for 30 s, and 72 °C for 1 min. The final products were sequenced and compared with the original template.

### 2.5. Genome Annotation and Phylogenetic Analyses

Genome annotation of the viral genome was performed using the Conserved Domain Database (CDD). The related virus reference sequences were downloaded from the NCBI non-redundant protein database. Multiple amino-acid sequences were aligned using the L-INS-i algorithm of MAFFT (version 7.490) [18] and trimmed using trimAl (version 1.2) [19], which was set by heuristic selection of the automatic method based on similarity statistics. Phylogenetic analysis and model selection were performed using IQ-TREE [20], with models chosen by the Bayesian information criterion (BIC) and 1000 bootstrap replicates.

## 3. Results

### 3.1. Overview of the Virome in H. erectus

HTS of the six *H. erectus* samples generated a total of 213,770,166 clean reads, which were assembled de novo. BLASTx identified 539 virus-associated contigs. By further screening and extension of these contigs, we finally identified three novel viruses, including HeAstV1 in five libraries (unhealthy = 3, normal = 2), Hippocampus erectus paramyxovirus 1 (abbreviated as HePmV1) in two libraries (unhealthy = 1, normal = 1), and Hippocampus erectus picornavirus 1 (abbreviated as HePcV1) in three libraries (unhealthy = 2, normal = 1) (Table 1 and Table S1). Therefore, all the three novel viruses were identified in both unhealthy and normal samples. In addition, a strain of NNV was also identified in one unhealthy sample but not in the remaining samples. However, the unhealthy group showed a higher viral diversity and abundance than the normal group, and the viral abundance was reflected by the reads per kilobase per million reads (RPKM) (Table 1 and Table S1).

### 3.2. Hippocampus erectus Astro-like Virus 1

HTS of the seahorse samples identified a contig of ~7000 nucleotides (nt) in length associated with the family *Astroviridae*. After further extension, a consensus sequence of 7114 nt was finally obtained with 4584 non-repetitive reads and a mean depth of 96.6 ± 21.7, which was tentatively named HeAstV1. To verify the viral genome obtained from HTS, we performed RT-PCR and Sanger sequencing, with the Sanger sequencing results consistent with that from HTS.

**Figure 1:**
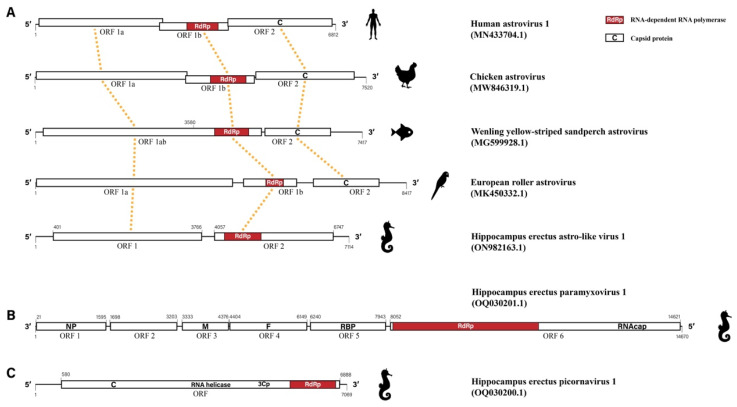
Viral genome structures of the three novel RNA viruses from *H. erectus*. (**A**) Genome diversity of representative astroviruses and Hippocampus erectus astro-like virus 1 (HeAstV1). The gene regions encoding RNA-dependent RNA polymerase (RdRp) and capsid proteins were highlighted. Homologous regions are connected with orange dotted lines. (**B**) Viral genome structure of Hippocampus erectus paramyxovirus 1 (HePmV1). (**C**) Viral genome structure of Hippocampus erectus picornavirus 1 (HePcV1).

The complete genome sequence of HeAstV1 consisted of 5′ and 3′ untranslated regions (UTRs) and two open reading frames (ORFs) of 3366 nt (ORF 1) and 2691 nt (ORF 2) in length, respectively (Figure 1A). ORF 1 encoded a polypeptide of 1121 aa from position 401 to 3766, which contained a serine peptidase at amino acid (aa) positions 540–601 and four potential transmembrane domains (TM) located at aa positions 110–119, 259–270, 325–339, and 373–382, respectively. It also contained a typical seven-base sliding sequence (AAAAAAC) of the astrovirus from positions 2567 to 2573 nt. The ORF2 starting from genome position 4057 to 6747 encoded a protein of 896 aa, which contained the RNA-dependent RNA polymerase (RdRp) from 4288 to 5121. However, no capsid proteins were found in ORF2 and other locations in the obtained viral sequences, either by CD-search or Pfam (Figure 1A).

Generally, ORF1a and ORF1b of typical viruses in the family *Astroviridae* were encoded by two separate ORFs, with ORF1b encoding RdRp. However, different astroviruses showed variations in the genome structure, especially in aquatic animals (Figure 1A). For example, the ORF1a and ORF1b of Wenling yellow-striped sandperch astrovirus found in fish were encoded by a single consecutive ORF—ORF1ab, with RdRp encoded by a gene region close to the 3′ terminal of ORF1ab. However, for HeAstV1, RdRp was encoded by ORF2. Therefore, emerging astroviruses revealed an increasing diversity in the genome structure of astroviruses (Figure 1A).

The first hit in the BLASTx output for HeAstV1 was the ORF1b of European roller astrovirus strain Er/SZAL5/HUN/2011 (MK450332.1), isolated from *Coracias garrulus,* with an aa identity of 49.49%. In addition, HeAstV1 shared 41.21% and 41.02% aa identity with chicken astrovirus isolates CAV/Belgium/4134_001/2019 (MZ367372.1) and CAstV/Chicken/CHN/2020/GD202013 (MW846319.1), respectively.

**Figure 2:**
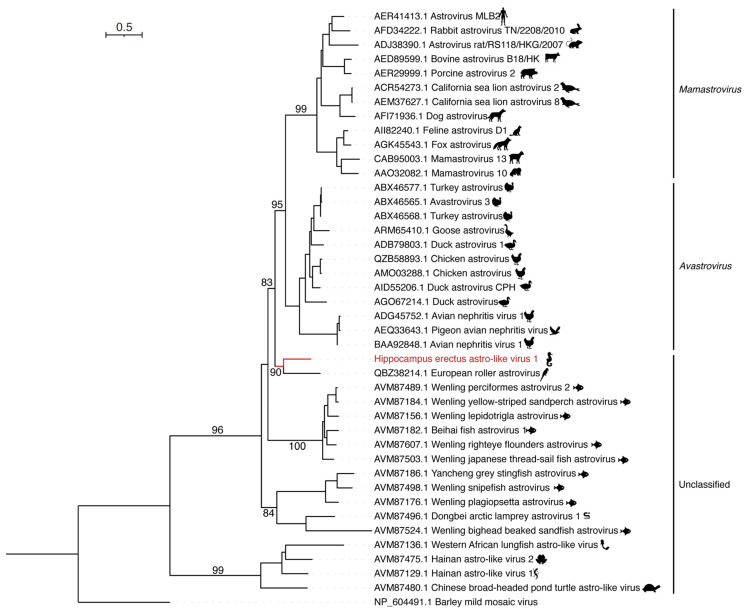
Phylogenetic analysis of the RdRp sequences of Hippocampus erectus astro-like virus 1 (HeAstV1) and representative astroviruses. The reference sequences were downloaded from GenBank and aligned using Mafft. Phylogenetic analysis was conducted using IQ-TREE, with the best-fit model LG + I + G4 and 1000 bootstrap replicates. Only bootstrap values > 80% are shown.

To investigate the phylogenetic relationships of HeAstV1, we performed a phylogenetic analysis of the RdRp sequences of representative and unclassified astroviruses and found that HeAstV1 did not fall within the two separate lineages of *Mamastrovirus* (MAstV) and *Avastrovirus* (AAstV) (Figure 2). Instead, it formed a separate lineage with European roller astrovirus. To our knowledge, this is the first report of an astrovirus in *H. erectus*.

### 3.3. Hippocampus erectus Paramyxovirus 1

A viral consensus of 14,670 nt in length consisting of 4504 non-repetitive reads with a mean depth of 46.0 ± 21.5 was identified. The first hit of BLASTx was Wenzhou Rattus losea jeilongvirus 2 (OM030338.1), with a 39.49% aa identity. Meanwhile, the hit list of BLASTx also contained many strains of Human respirovirus 1, with an aa identity of 39.30%. As both viruses were members of the family *Paramyxoviridae*, we tentatively named the novel virus HePmV1.

The complete genome sequence of HePmV1 consisted of six ORFs encoding nucleocapsid (NP), matrix (M), fusion (F), haemagglutinin-neuraminidase (HN), RdRp, and mRNA capping enzyme (CE), which is roughly consistent with other paramyxoviruses in terms of genome structure. However, the paramyxovirus P/V phosphoprotein C-terminal, an essential component of the RNA polymerase transcription and replication complex that is supposed to be present in ORF2, was not identified in HePmV1 (Figure 1B).

**Figure 3:**
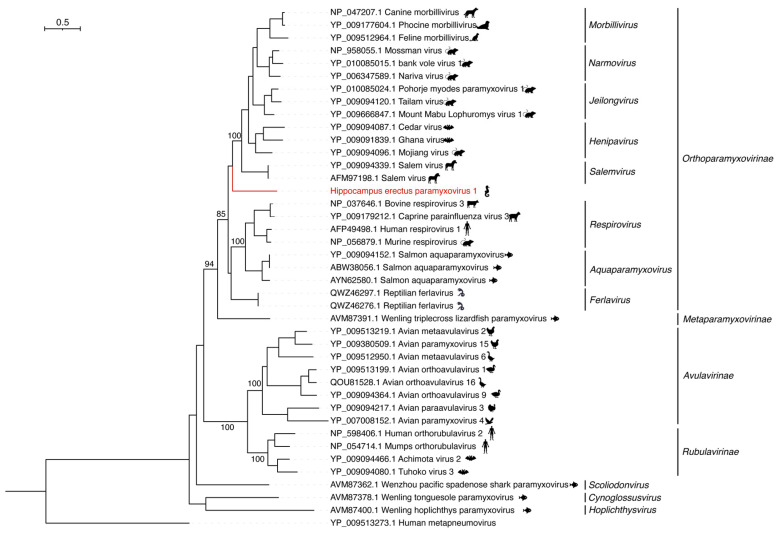
Phylogenetic analysis of the RdRp sequences of HePmV1 and representative paramyxoviruses. Phylogenetic analysis was performed using IQ-TREE, with the best-fit model LG + F + R5 and 1000 bootstrap replicates. Only bootstrap values > 80% were shown.

Although HePmV1 showed approximately 45.55% aa identity in the RdRp to Salem virus (NC_025386.1) from the genus *Salemvirus*, family *Orthoparamyxovirinae* (Figure 3), HePmV1 did not fall within the genus *Salemvirus*. Alternatively, it formed a separate branch, representing a new member of the subfamily *Orthoparamyxovirinae*.

### 3.4. Hippocampus erectus Picornavirus 1

A picornavirus with a near-complete genome named HePcV1 was identified from three pools, with a high expression level in one of the unhealthy samples (Table 1). The genome length of HePcV1 was 7069 nt, which consisted of 5′ and 3′ UTRs as well as a single ORF of 2102 aa from position 580 to 6888. HePcV1 shared the highest identity with Human parechovirus 1 (KY645963.1), with an aa identity of 32.07% by BLASTx. HePcV1 was predicted to encode two capsid proteins, an RNA helicase, a 3C cysteine protease (3Cp), and RdRp (Figure 1C).

**Figure 4:**
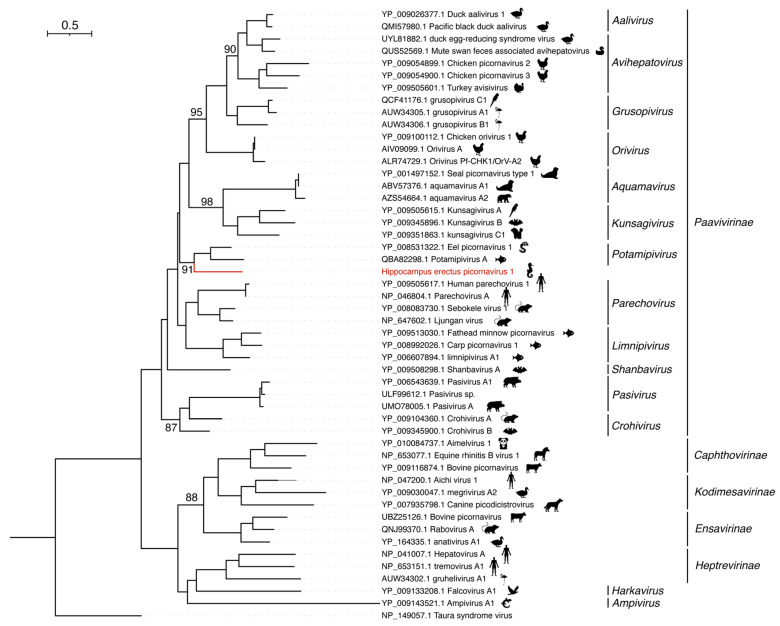
Phylogenetic analysis of the RNA-dependent RNA polymerase (RdRp) sequences of Hippocampus erectus picornavirus 1 (HePcV1). Phylogenetic analysis was performed using IQ-TREE, with the best-fit model LG + I + G4 and 1000 bootstrap replicates. Only bootstrap values > 80% were shown.

HePcV1 is phylogenetically positioned within the subfamily *Paavivirinae*, family *Picornaviridae* (Figure 4). In addition, it clustered together with Eel picornavirus 1 (NC_022332.1), identified from *Anguilla anguilla* and Potamipivirus A (MK189163.1) from *Gasterosteus aculeatus*, both of which belonged to the genus *Potamipivirus*. However, HePcV1 showed only an approximately 33.74% aa identity to the Eel picornavirus 1 in the polyprotein, and we therefore propose that HePcV1 might represent a new genus of the family *Paavivirinae*.

### 3.5. Nervous Necrosis Virus

We identified fourteen contigs in the HTS data of a hippocampal sample, which were highly similar to NNV from different hosts. All of these contigs had substantially higher RPM than the three novel viruses identified (Table 1). After assembly, we obtained two segments of 3024 nt (RNA1) and 1362 nt (RNA2) in length, respectively. The BLASTn analysis showed that RNA1 shared the highest nucleotide identity of 98.94% with murray cod nervous necrosis virus (MW729335.1) in the RdRp gene, and RNA2 shared a nucleotide identity of 99.41% with pearl gentian grouper nervous necrosis virus (MG637439.1) in the coat protein gene. In addition, it shared a nucleotide identity of 99.12% with a partial sequence of an NNV strain (seahorse nervous necrosis virus) from *H. abdominalis* [21]. Conserved domain prediction revealed that RNA1 encoded RdRp with a Methyltransferase and RNA2 encoded a coat protein, which was in agreement with the typical genome structure of NNV.

## 4. Discussion

With the increasing attention to marine resources and the development of meta-transcriptomic sequencing, more and more marine viruses have been identified in recent years. However, there is still a paucity of studies on the virome of seahorses, one of the most important marine economic animals. In this study, we used meta-transcriptomic sequencing to explore the viruses in *H. erectus*.

We finally identified three novel RNA viruses, including HeAstV1, HePmV1, and HePcV1, and a strain of nervous necrosis virus from *H. erectus*. With the exception of NNV, all three novel viruses were found in more than two samples. A comparison of the viromes between unhealthy and normal seahorses is helpful in identifying a potential causative agent and viruses with a higher abundance in the diseased group are more likely to be potentially high-risk viruses for seahorses. However, additional studies, such as infection experiments, electron microscopy, and in situ hybridization, are required to confirm whether these novel viruses cause the disease in *H. erectus*.

Of particular note was HeAstV1, which was found in both batches of hippocampal samples, with a higher abundance in unhealthy samples than in healthy samples. Currently, *Astroviridae* includes two genera, MAstV and AAstV, which infect mammalian (human, bovine, feline, dolphin, mink, sea lion, and porcine) and avian species (chicken, duck, turkey, and goose), respectively [22,23,24,25]. A few astro-like viruses have also been reported in aquatic vertebrates in recent years, including Beihai fish astrovirus, Wenling gobies fish astro-like virus, and Western African lungfish astro-like virus [26]. However, no astrovirus infecting seahorses has been previously documented. Notably, HeAstV1 is grouped together with European roller astrovirus. However, both viruses differed from other astroviruses in terms of genome structure. This revealed an increasing genomic diversity than previously expected. Furthermore, both paramyxovirus and picornavirus have a wide host range, including mammals, avian species, and fish [27,28]. In this study, we identified HePmV1 and HePcV1 in the hippocampus, which further expands the host range of paramyxovirus and picornavirus.

In addition, we also found NNV in one diseased *H. erectus*. NNV is the causative agent of viral nervous necrosis (VNN) in marine fish, and its hosts are mainly groupers, sea bass, and flatfish. NNV is one of the major pathogenic viruses for worldwide fisheries due to the high mortality and broad host range [29,30]. Recently, it has also been reported to kill *H. abdominalis* [21]. However, to our knowledge, this is the first report of NNV in *H. erectus*, suggesting the expanding host range of NNV. Whether NNV is related to the disease in *H. erectus* requires further investigation.

## 5. Conclusions

In this study, we described three novel RNA viruses from *H. erectus*, including HeAstV1, HePmV1, and HePcV1, as well as a strain of nervous necrosis virus, which provided novel insights into genome diversity and the cross-species transmission of viruses in seahorses. In addition, this study also revealed a greater diversity in the genome structure, as implicated by HeAstV1 of the *Astroviridae* family. However, further investigation is required to determine which virus is associated with the disease.

## Figures and Tables

**Table 1:** Viral abundance in *Hippocampus erectus*.

Virus	Sample	Batch	Group	RPKM ^a^
Hippocampus erectus astro-like virus 1	FRRL190067409	201907	Normal	8.92
	FRRL190067410		Normal	- ^b^
	FRRL190067412		Unhealthy	15.68
	FRRL190067411		Unhealthy	13.72
	FRRL192024264	201911	Normal	1.79
	FRRL192024267		Unhealthy	4.09
Hippocampus erectus paramyxovirus 1	FRRL190067409	201907	Normal	16.94
	FRRL190067410		Normal	- ^b^
	FRRL190067412		Unhealthy	- ^b^
	FRRL190067411		Unhealthy	7.61
	FRRL192024264	201911	Normal	- ^b^
	FRRL192024267		Unhealthy	- ^b^
Hippocampus erectus picornavirus 1	FRRL190067409	201907	Normal	- ^b^
	FRRL190067410		Normal	- ^b^
	FRRL190067412		Unhealthy	- ^b^
	FRRL190067411		Unhealthy	166.02
	FRRL192024264	201911	Normal	17.79
	FRRL192024267		Unhealthy	38.56
nervous necrosis virus	FRRL190067409	201907	Normal	- ^b^
	FRRL190067410		Normal	- ^b^
	FRRL190067412		Unhealthy	6610.70 (RdRp ^c^) 6125.40 (cp ^d^)
	FRRL190067411		Unhealthy	- ^b^
	FRRL192024264	201911	Normal	- ^b^
	FRRL192024267		Unhealthy	- ^b^

^a^ RPKM: reads per kilobase per million reads; ^b^ -: not detected; ^c^ RdRp: RNA-dependent RNA polymerase; ^d^ cp: coat protein.

## Data Availability

Transcriptome data are available under NCBI BioProject PRJNA857333.

## Supplementary Materials

The following supporting information can be downloaded at: https://www.mdpi.com/article/10.3390/v15030772/s1, Table S1: Diversity of viruses in hippocampus; Table S2: Primers used for the amplification and sequencing of HeAstV1.
