# Supplementary material for: Meta-Transcriptomic Analysis Reveals Novel RNA Viruses in Hippocampus erectus
Source: Viruses. 2023 Mar 17;15(3):772. doi: 10.3390/v15030772 (PMC10053862; doi:10.3390/v15030772)
Supplement: Supplementary file 1 [file viruses-15-00772-s001.zip › Table S2.pdf]

Table S2 Primers used for amplification and sequencing of the HeAstV1.

Primer	Sequence 5'-3'	Position 5'-3' (nt)
HeA-1F	CGGAGCTAACCGCAGATGT	3-21
HeA-1R	TGTTGCCCAAATCCTCAGGG	500-481
HeA-2F-O	ACATCATACGTGCTTCCCGA	287-306
HeA-2R-O	GGCAATGCTCTTACCGACCT	1000-981
HeA-2F-I	AATTGGCTTCTTTAAGGACAGGT	306-328
HeA-2R-I	CAGCAGACAAATGCCGTCAC	955-936
HeA-3F	TGACAGTGGTTGTCGTATTGAGA	853-875
HeA-3R	CAAAGGCAAAAGTGGCGTCC	1391-1372
HeA-4F-O	AGGCTTTGATCCGACCAGTG	1156-1175
HeA-4R-O	ATCATGATCGAGGACGTGGC	1870-1851
HeA-4F-I	TTGCGGTTGCCCTTCACTAT	1197-1216
HeA-4R-I	TGGTGATCAACTTTCCGCGA	1847-1828
HeA-5F-O	GTGTGCCTAGCGCCATTTTT	1710-1729
HeA-5R-O	AGCAAGTTCGTCTCGCTTCA	2356-2337
HeA-5F-I	GTCATACCGTGTGTCCCCTT	1753-1772
HeA-5R-I	CTTCCTTGGTGGCTTTGTCG	2333-2314
HeA-6F-O	CCAAGAAGGGATACGCTGGG	2013-2032
HeA-6R-O	TCGTCACTGGCTACATCTTCG	2730-2710
HeA-6F-I	GGGACCTACTGTAACGCCAG	2030-2049
HeA-6R-I	ATCGTTTGTCAGCGAGGTGG	2693-2674
HeA-7F	ACTTCAGAGTCCGCAGACCA	2580-2599
HeA-7R	GAGTGCGTTGGCTTGTTGTA	3055-3036
HeA-8F-O	AACATGAGGCAGATAGGCGT	2976-2995
HeA-8R-O	TGCTGCCGGGGTTTTCTTTA	3716-3697
HeA-8F-I	GAGGCAGATAGGCGTCAAAT	2981-3000
HeA-8R-I	GCAGTTTTTATCACCCACGC	3685-3666
HeA-9F-O	GCATTGTGGCGAACCTACAC	3529-3548

HeA-9R-O	AGACCTTGGTCTTCCAGCAG	4268-4249
HeA-9F-I	TTGTGGCGAACCTACACCAG	3532-3551
HeA-9R-I	TCCAGCAGATCCTTTTCGGT	4256-4237
HeA-10F	CTACCAGCTAGCCCTTGACAC	4107-4127
HeA-10R	ACCAATCGAGGAGGACTTCG	4687-4668
HeA-11F-O	GGCTACCCGAAAAAGCAGAA	4210-4229
HeA-11R-O	GTACGAATGTCTTCTCGGCG	4948-4929
HeA-11F-I	GGCTACCCGAAAAAGCAGAAG	4210-4230
HeA-11R-I	CCGGTCGTCACCGTAACATA	4914-4895
HeA-12F-O	GTTAACACCTGCCTCGTTGC	4798-4817
HeA-12R-O	TGTCGTCAGGTATCGCACTG	5482-5463
HeA-12F-I	TGCCTACGAAACGTCTTGCT	4815-4834
HeA-12R-I	CGTCAGGTATCGCACTGTGT	5479-5460
HeA-13F	GAGTACCGCGACTACGTGTT	5245-5264
HeA-13R	TCTGGAAAGCGCCATAAGCA	6250-6231
HeA-14F	TTACCATCCGAACCACCGAT	6086-6105
HeA-14R	TTTCGAGGACGATTCCCACC	6990-6971
HeA-15F	TCTGAATGAGGGCAACGTCC	6132-6151
HeA-15R	TCGAGGTAACGTGTGTACATT	7084-7064
